# Leflunomide inhibits the apoptosis of human embryonic lung fibroblasts infected by human cytomegalovirus

**DOI:** 10.1186/2047-783X-18-3

**Published:** 2013-02-01

**Authors:** Ren Qi, Zeng Hua-Song, Zeng Xiao-Feng

**Affiliations:** 1 The Department of Pediatric, Allergy, Immunology and Rheumatology,Guangzhou Women and Children's Medicial Center, Guangzhou Medical University and First Clinical Medical College, Jinan University, China; 2 The Department of Rheumatology, Peking Union Medical College Hospital, Chinese Academy of Medical Sciences, China

**Keywords:** Leflunomide, Human cytomegalovirus, Human embryonic lung fibroblast, Apoptosis

## Abstract

**Background:**

The immunomodulatory drug leflunomide (LEF) is frequently used for treating human cytomegalovirus (HCMV), but its antiviral mechanism is still unclear. In this study,we therefore investigated the effects of the active LEF metabolite A771726 on the HCMV lifecycle in human embryonic lung fibroblasts. We clarified the mechanism of LEF antiviral infection, and provide a new way to treat immune dysfunction patients with HCMV infection.

**Methods:**

The experiment was divided into four groups: the control group, the HCMV group, the ganciclovir + HCMV group as well as the LEF + HCMV group. MTT was usedfor assessment of the cell inhibitory rate. Apoptosis was measured by staining with fluorescein isothiocyanate Annexin V and propidium iodide. Statistical significance was determined by paired *t-*test using SPSS software.

**Results:**

The results of the study showed that cell proliferation was significantly inhibited by HCMV at 24 hours and 48 hours. With increasing HCMV concentration, the value-added inhibition of the cells was significantly decreased compared with the control group, and was statistically significant (*P* <0.01). Ganciclovir can increase proliferation of cellsinfected with HCMV; compared with the control group it was statistically significant (*P* <0.05). Meanwhile, with LEF treatment cell proliferation was significantly improved at 24 hours and 48 hours, with statistical significance (*P* <0.05). The apoptosis rate of human embryonic lung fibroblasts infected with HCMV increased significantly at 24 hours, 48 hours and 72 hours, and as time goes on the apoptosis rate increases statistically significantly (*P* <0.01) compared with the control group The apoptosis rate of theHCMV infection group decreased by adding LEF,and was statistically significant (*P* <0.05).

**Conclusions:**

In this studywe show that LEF is an exciting new drug for cytomegalovirus infection. LEF significantly inhibited HCMV infection-induced apoptosis and proliferation, playing an important role in the treatment of patients infected by HCMV. In this study we explored the potential usefulness of LEF for cytomegalovirus infection and found it to be a cost-effective new treatment for cytomegalovirus infection that deserves further study.

## Background

Leflunomide (LEF) is an immunosuppressive drug used clinically for the treatment of transplantation patients. Interestingly, besides its immunosuppressive activities, LEF can interfere with the replication of many viruses. Human cytomegalovirus (HCMV) belongs to the β subfamily of the herpes virus, and is the most common pathogen of utero and postnatal infection, which can cause stillbirth, fetal malformation, miscarriage, developmental retardation and abnormal liver function. HCMV is the major pathogen of congenital malformations, growth retardation of the nervous system, hearing and visual impairment, blood system diseases, infant hepatitis and other serious diseases. HCMV infections are also common causes for people who have low immune function, such as organ transplant or AIDS patients. HCMV can replicate in the human embryonic lung fibroblasts. The mechanism of apoptosis inducted by HCMV infection is not clear. Another study showed that HCMV infection can cause clinical disease and apoptosis, which are closely related to each other [1]. As cell physiological death, apoptosis play an important role in reducing aging and maintaining the balance of the body, but when the cell undergoes abnormal apoptosis it can cause a variety of diseases. In recent years, the study of HCMV and cell apoptosis has made great progress. The relationship between HCMV and apoptosis is more complexHCMV can inhibit cell apoptosis or promote apoptosis in the infection body [2]. The study found that the early stages of HCMV infection manifested as inhibition of apoptosis, which provided sufficient time for HCMV in cell proliferation [3]. With extension of the time of infection, however, the inhibition of apoptosis was changed by the promotion of apoptosis, which can cause loss of cell function and a number of pathological and clinical diseases. The higher the dose of HCMV infection, the sooner the cell mediated apoptosis [4]. LEF is a novel immunosuppressant, formation of the active metabolite A771726 playing a role in the body. Treatment with LEF A771726 may counteract the riskof enhancing HCMV replication in infected patients.

## Methods

### Cell culture

The cells were obtained from Zhongshan University(Ethical approval was given by the medical ethics committee of Sun Yat-sen University with the following reference number: SYXK20070081), and were maintained in 1640 medium, supplemented with 10% fetal calf serum and 1 × 10^5^ U/l penicillin, and treated with the LEFHCMV and ganciclovir (GCV). The experiment was divided into four groups: the control group, the HCMV group, the GCV + HCMV group, as well as the LEF + HCMV group. The control group included 0.02% DMSO RPMI 1640, the HCMV group included HCMV, the GCV + HCMV intervention group included GCV and HCMV, and the LEF + HCMV intervention group included LEF and HCMV. Cells were then incubated at 37°C with a humidified atmosphere of 5% CO_2 _in air. The above experimentwas performed three times.

### Assessment of inhibitory rate

MTT was used to assessthe cell inhibitory rate. Cell suspensions were placed in triplicate in 96-well culture plates at 10,000 cells/100 μl per well. Cells were incubated for 96 hours at 37°C and 100 μl MTT solution was added to each well. Cells were then incubated at 37°C for a further 4 hours. The plates were read on an automatic plate reader at a wavelength of 570 nm and the background absorbance measured at 690 nm was subtracted.

(1) $$\mathrm{Inhibitory}\;\mathrm{rate}\left(\%\right)=(\mathrm{experimental}\;\mathrm{group}/\mathrm{control}\;\mathrm{group})\times 100.$$

The above experiments were repeated three times.

### Assessment of apoptosis

Cells grown under growth-restrictive conditions were plated at a density of 1 × 10^6^/ml and allowed to attach to tissue culture plates for 24 hours. Cells then were incubated with medium that contained 10% fetal bovine serum in the presence or absence of HCMV. LEF or GCV was added to the medium after 1 hour. The percentage of apoptotic cells was assessed, and cells were harvested at 24 hours and 48 hours. Apoptosis was measured by staining with fluorescein isothiocyanate–Annexin V and propidium iodide ((Sigma-Aldrich, Guangzou, Guangdong, China)). We then added 500 μl cell suspension liquid to 5 μl fluorescein isothiocyanate–Annexin V and 5 μl propidium iodide, avoiding light at 4°C for 10 minutes. Absorbance was recorded at an excitation wavelength of 448 nm, collecting 10,000 cellsfor each sample specimen. The cell apoptosis rate was then measured using the relevant software. The experiment was repeated three times.

### Statistical analysis

Statistical significance of differences in measured variables between controls and treated groups was determined by paired *t* test using SPSS for Windows V.13.0 (SPSS, Chicago, IL, USA). ≤0.05 was considered statistically significant.

## Results

### Inhibitory effect of leflunomide on human embryo lung cells infected with HCMV

**Figure 1:**
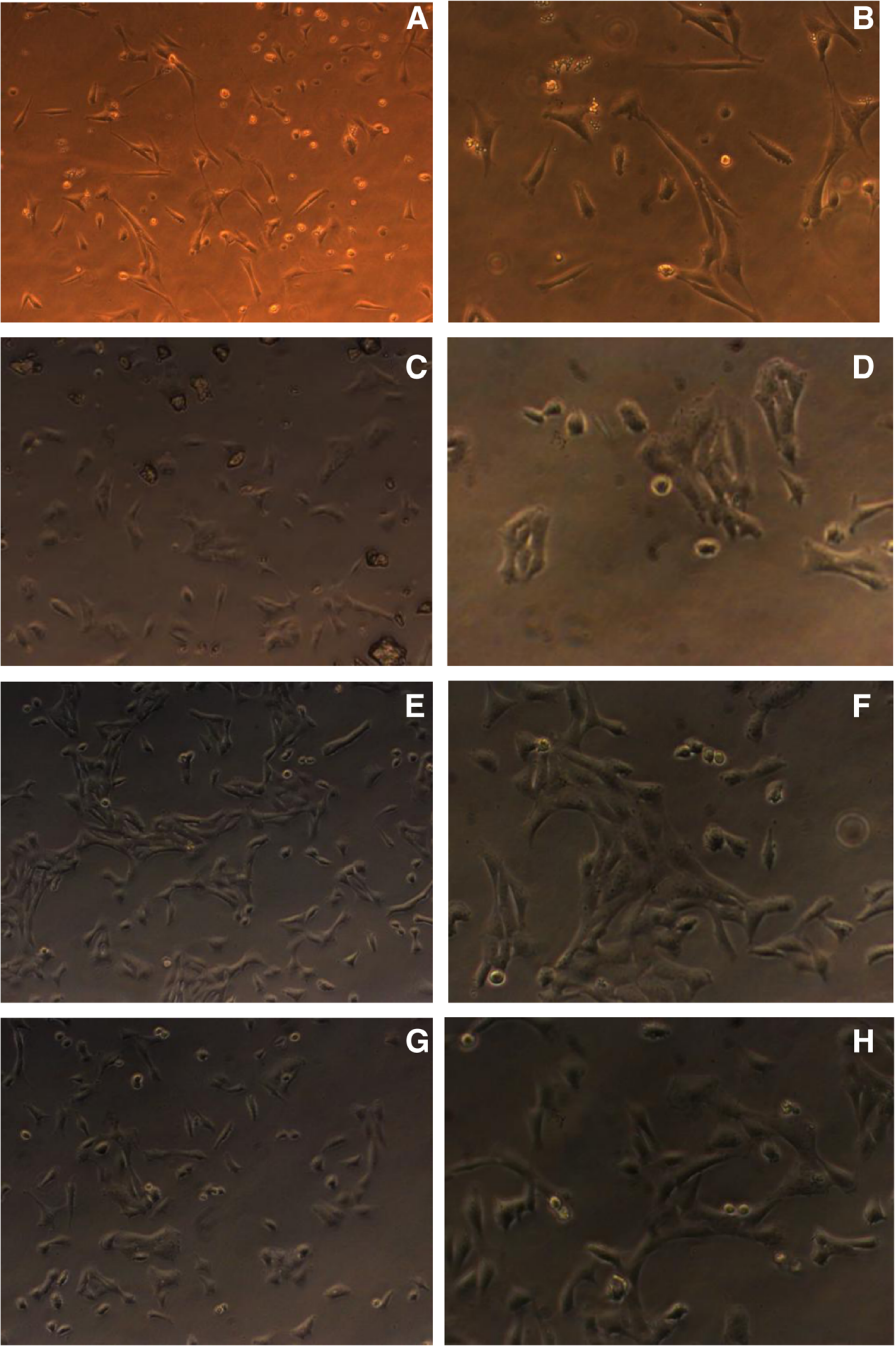
**Cell changes in cell morphology at 24 hours and 48 hours.** (**A**) Control group (normal human embryonic lung fibroblast at 24 hours, microscope × 200). (**B**) Control group (normal human embryonic lung fibroblast at 48 hours, microscope × 400). (**C**) Human cytomegalovirus (HCMV) group (human embryonic lung fibroblast infected with HCMV at 24 hours, microscope × 200). (**D**) HCMV group (human embryonic lung fibroblast infected with HCMV at 48 hours, microscope × 400). (**E**) Ganciclovir (GCV) + HCMV group (GCVadded to the human embryonic lung fibroblast infected with HCMV at 24 hours, microscope × 200). (**F**) GCV + HCMV group (GCV added to the human embryonic lung fibroblast infected with HCMV at 48 hours, microscope × 400). (**G**) Leflunomide (LEF) + HCMV group (LEF added to human embryonic lung fibroblast infected with HCMV at 24 hours, microscope × 200). (**H**) LEF + HCMV group (LEF added to the human embryonic lung fibroblast infected with HCMV at 48 hours, microscope × 400).

**Figure 2:**
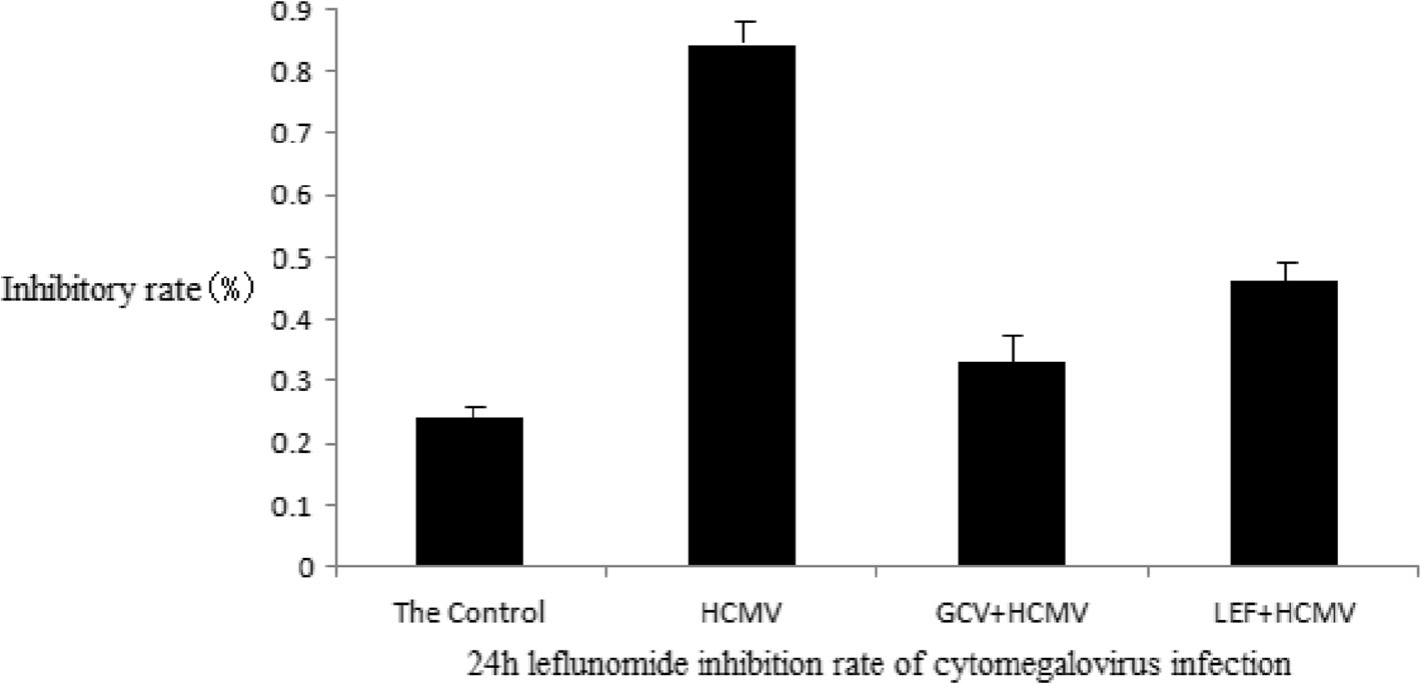
Leflunomide inhibition rate of cytomegalovirus infection in each group at 24 hours.

**Figure 3:**
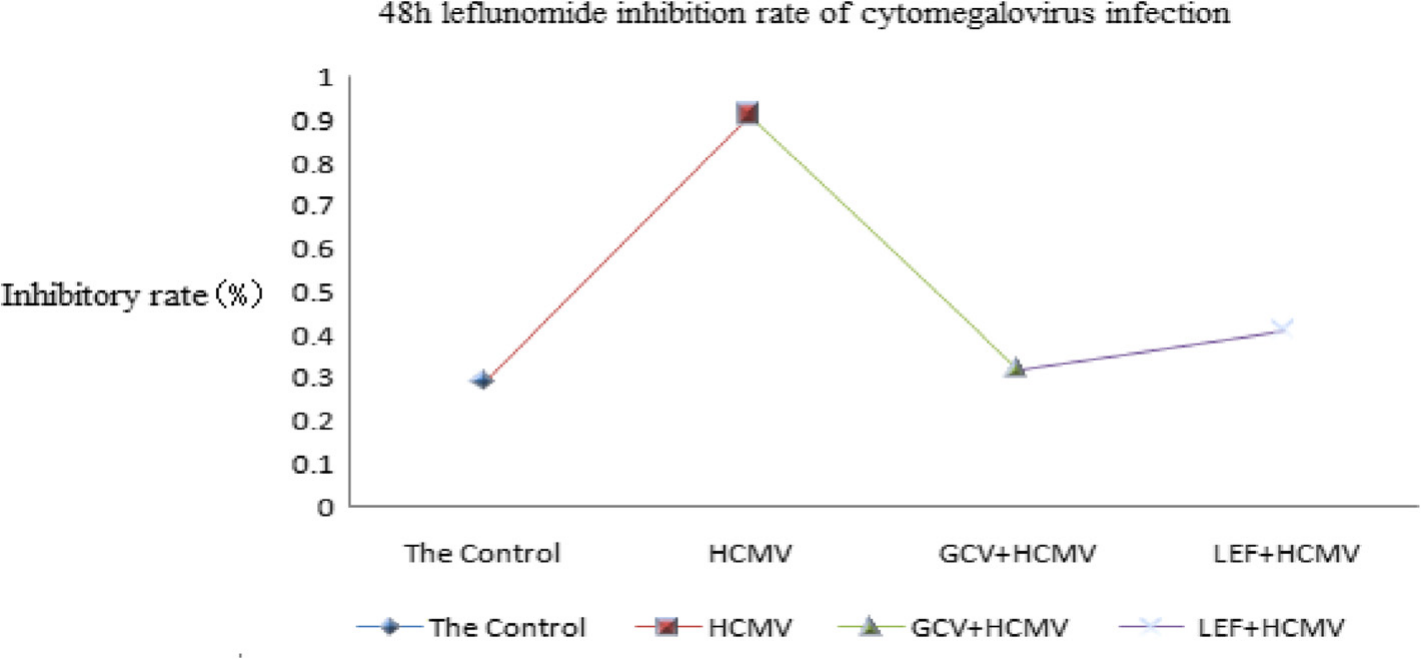
**Leflunomide inhibition rate of cytomegalovirus infection in each group at 48 hours.** In this study, we found that the leflunomide (LEF) inhibition rate of cytomegalovirus (CMV) infection has obvious effects at 24 hours and 48 hours. Compared with the control group, the CMV infection group was statistically significant (*P* <0.05).

The results of this study showed that cell proliferation was significantly inhibited by HCMV at 24 hours and 48 hours (see Table 1). With increasing HCMV concentration, the value-added inhibition of cells was significantly decreased compared with the control group andwas statistically significant (*P* <0.01). GCV can increase proliferation of cells infected with HCMV, statistically significantly (*P* <0.05) compared with the control group. Meanwhile, with LEF treatment the cell proliferation was significantly improved at 24 hoursand 48 hours, with statistical significance (*P* <0.05). Results are shown in Figures 1, 2 and 3.

**Table 1:** Inhibitory rate of leflunomide on human embryo lung cells

**Group**	***n***	**24 hours**	**48 hours**	**Inhibitory rate (%)**	
				**24 hours**	**48 hours**
Control	6	0.523 ± 0.045	0.813 ± 0.078		
LEF1	6	0.43 ± 0.062*	0.572 ± 0.061*	39.7	47.1
LEF2	6	0.568 ± 0.045*	0.84 ± 0.028*	21.4	30.8
LEF3	6	0.654 ± 0.032*	0.932 ± 0.031*	8.3	12.5

LEF, leflunomide. compared with the control; *p <0.05, *P <0.05.

### Effect of leflunomide on the proliferation of human embryonic lung cells

The final concentration of LEF was divided into three separate groups 40 g/ml, 20 g/ml, and 5 g/ml. The effect of human fetal lung cell proliferation at the different doses of LEF was still significant (*P* <0.05).

### Apoptosis detection

**Figure 4:**
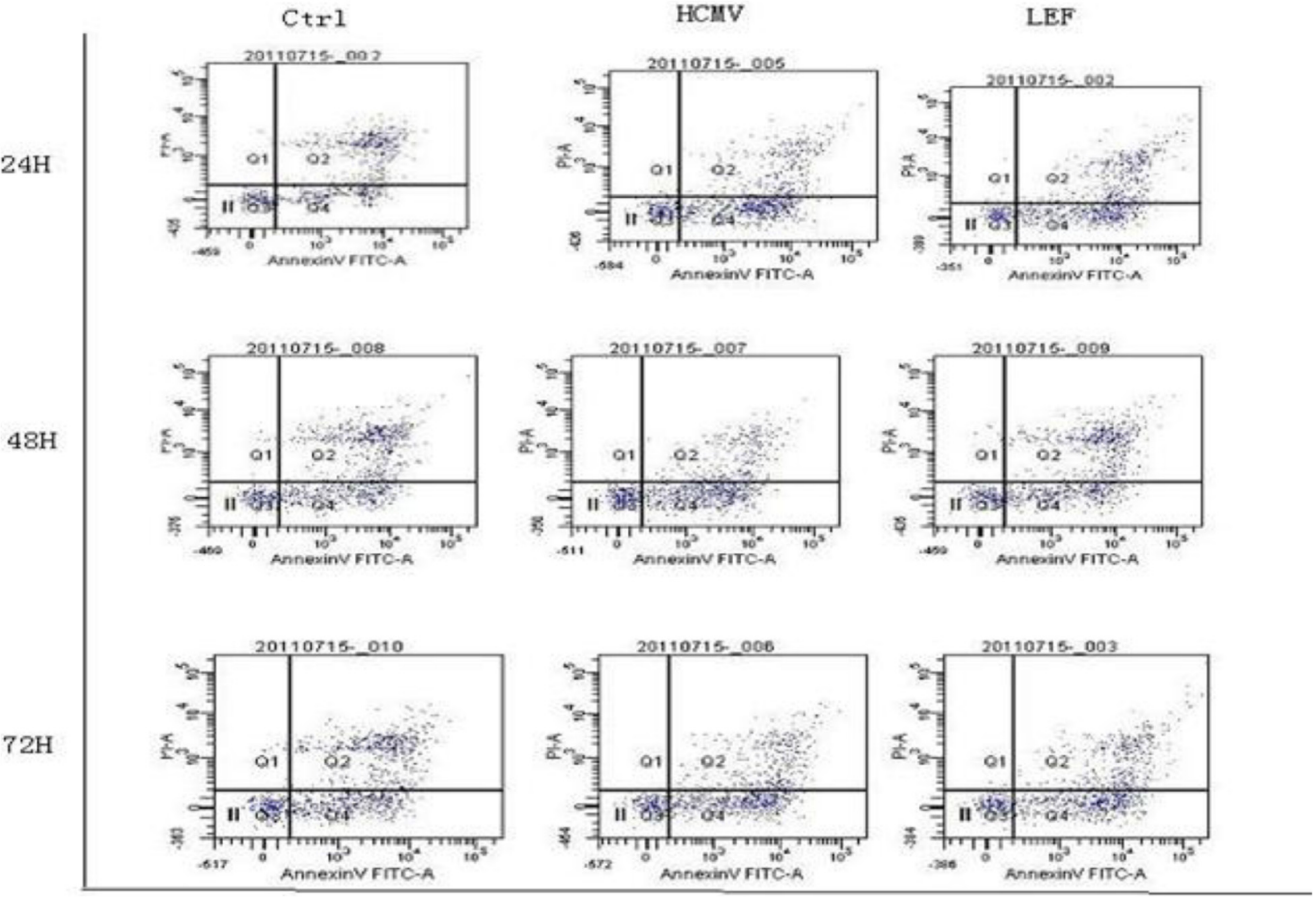
Apoptosis in each group at 24 hours, 48 hours and 72 hours.

**Figure 5:**
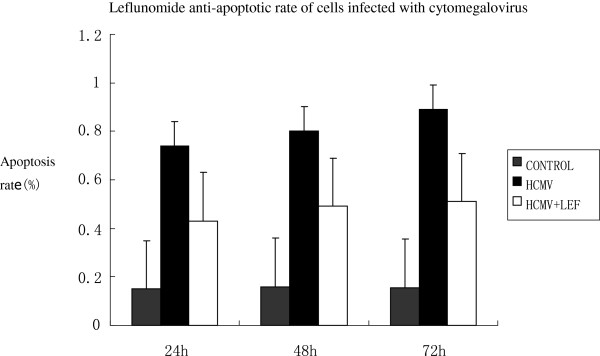
**Apoptosis rate of cells at 24 hours, 48 hours and 72 hours.** At 24 hours, 48 hours and 72 hours, leflunomide (LEF) has a significant anti-human cytomegalovirus (anti-HCMV) infection. Compared with the control group, the apoptotic effect of cytomegalovirus (CMV) infectionwas significant (*P* <0.05).

The apoptosis rate of human embryonic lung fibroblasts infected with HCMV increased significantly at 24 hours, 48 hours and 72 hours, and as time goes on the apoptosis rate increases; compared with the control group it was statistically significant (*P* < 0.01). The apoptosis rate of the HCMV infection group decreased by adding LEF, and was statistically significant (*P* <0.05). See Figures 4 and 5.

## Discussion

Cytomegalovirus (CMV) is one of the most important infections in the immunosuppressed patient. CMV disease often remains a life-threatening complication. In addition, studies have suggested a role for this virus as a contributing factor in allograft rejection. In the absence of any preventative therapy, CMV infection occurs in approximately 30 to 75% of transplant recipients, with an incidence of CMV disease between 8 and 80%. CMV infection may cause indirect effects, including acute and chronic re-infection, increased risk of cardiac complications, diabetes, and even death. Several drugs have been developed and approved for CMV treatment.

Apoptosis is programmed cell death, and is a multicellular animal life activity through gene regulation. Apoptosis mechanisms and the relationship of apoptosis with disease has become a hot topic in the field of biomedical research. Early stages of HCMV infection can block the apoptosis pathway to ensureviral protein synthesis, assembly and replication, resulting in persistent infection. HCMV infection increases the host cell apoptotic loss of cell function, leading to clinical symptoms. HCMV occurs mainly through the endogenous pathway to promote apoptosis [5], and p53 is an apoptosis regulatory factorthat plays an important role.

LEF is an inhibitor of protein kinase activity, and is an immunosuppressive drug used in rheumatoid arthritis and organ transplantation. Many reports have described the benefit of LEF for CMV infection and clinical use [6,7]. LEFis currently under discussion for use as an antiviral substance in the clinic, because of its inhibitory effects on several viruses. LEF is cheaper and is easily given orally compared with other antivirals. LEF works via a novel mechanism and also has immunosuppressive properties. This study also found that LEF was effective in CMV infection. Given these considerations we believe that LEF is an exciting new drug for CMV infection. An American study showed that 88% of kidney transplant recipients suffered CMV infection [8]. Many studies have shownthat the meaningful value of LEF lies in its organ transplantation, bone marrow transplantation and hematopoietic stem cell transplant recipients with CMV infection [9]. In addition, animal experimentsof CMV confirmed that after treatment usingLEF the intracellular levels were significantly decreased by 75 to 99% [10]. LEF treated active rheumatoid arthritis patients with the HLA-DRB1 gene [11]. Recent studies have shown that LEF can reduce CMV infection of transplant recipients [12]. In addition, rheumatoid arthritis, systemic lupus erythematosus, glomerular diseases, skin diseases and other therapeutic areas also achieved good results. It is encouraging that LEF in the treatment of HCMV infection has also been a widespread concern [13]. The first report stated that LEF has anti-HCMV effects in renal transplant recipients. Recent clinical studies have shown that LEF has a better therapeutic effect on HCMV infection disease after renal transplantation and reduces viremia, promotes the involvement of organ function recovery, and increases sensitivity to GCV in mice with HCMV infection [14-16]. LEF has a significant effect on refractory retinitis after allogeneic bone marrow transplantation in drug-resistant HCMV infection [17-19]. Some studies have shown that the LEF has a good effect on the stubborn resistance of HCMV infection [20,21].

In this study, we observed that cell proliferation was significantly inhibited by HCMV at 24 hours and 48 hours. With increasing HCMV concentrations, the value-added inhibition of cells was significantly decreased compared with the control group, and was statistically significant (*P* <0.01). Meanwhile, treatment with LEF significantly improved cell proliferation at 24 hours and 48 hours, with statistical significance (*P* <0.05). The effect of human fetal lung cell proliferation at the different doses of LEF was still significant (*P* <0.05). In addition, the apoptosis rate of human embryonic lung fibroblasts infected with HCMV increased significantly at 24 hours, 48 hours and 72 hours, and as time goes on the apoptosis rate is increased; compared with the control group it was statistically significant (*P* <0.01). The apoptosis rate of the HCMV infection group decreased by adding LEF, and was statistically significant (*P* <0.05). This finding confirmed that LEF is an effective new treatment for CMV infection.

## Conclusions

The search for newer more cost-effective treatments for infectious diseases remains a challenge. In this study, we believe that LEF is an exciting new drug for CMV infection. The future is likely to be first-line or second-line drug treatment for organ transplantation. AIDS immunocompromised patients infected with HCMV also rely on more clinical evidence, but our study showed that LEF significantly inhibited HCMV infection-induced apoptosis and proliferation, playing a important role in the treatment of patients infected by HCMV. In this study, we explored the potential usefulness of LEF for CMV infection and found that it to be a cost-effective new treatment for CMV infection that deserves further study.

## Abbreviations

CMV: Cytomegalovirus; GCV: Ganciclovir; HCMV: Human cytomegalovirus; LEF: Leflunomide; MTT: 3-(4,5-dimethylthiazol-2-yl)-2,5-diphenyltetrazolium bromide.

## Competing interests

The authors declare that they have no competing interests.

## Authors’ contributions

Z-HS participated in the design of the study. RQ performed the statistical analysis, collected the data and drafted the manuscript. All authors read and approved the final manuscript.
